# Chicago Biological Society

**Published:** 1881-06

**Authors:** 


### ARTICLE IV.

THE CHICAGO BIOLOGICAL SOCIETY.

Stated meeting May 4, 1881. Dr. W. S. Haines, President, in the chair.

Dr. Roswell Park read a paper on the

### SURGICAL ANATOMY OF THE SHEATHS OF THE PALMAR TENDONS.

Considering the frequency of injuries about the hands, one is disappointed to find how its anatomy has been poorly described by English anatomists. The best description of the Palmar Tendons has been written by Gosselin, and in this country by Connor, of Cincinnati.

After giving the anatomy of the tendons as commonly described, Dr. Park referred to the palmar bursa in which they glide. This bursa generally extends to the thumb and little finger, but the three other fingers have a bursa by themselves. This explains how phlegmonous inflammations of the thumb or little finger will rapidly extend through that synovial sac to the wrist and to the fore-arm. This bursa does not contain clear synovium, but cellular fibers also. It is best demonstrated by inflating it through its origin at the wrist. In some exceptional cases the thumb or the little finger have a separate bursa.

In cases of deep-seated inflammations in the hand, hot applications should be used, and as soon as suppuration has taken place the patient should be anæsthetized, the parts carefully dissected, cutting longitudinally over the middle of the metacarpal phalanges, and letting out the pus.

EXHIBITION OF SPECIMENS.

Dr. Park exhibited before the Society the various joints of the human body with all the ligaments attached, and perfectly flexible and odorless. They had stood exposed to the air for six months, and they were as soft and elastic as in fresh subjects. All the members present, some of whom were graduates of European colleges, admitted that they had never seen anything of the kind—it was a wonderful achievement in anatomical preparations. The sheaths of the tendons, with some of the flexor muscles, were preserved in the same manner. These preparations, at first perfectly white, had become yellowish-brown, but Dr. Park thought that was the only change that they could undergo, and that they would remain in the present state for years. His process of preparation was this:

The joints were dissected soon after death, then soaked in water for three weeks. They were now soaked in the following solution:

| | | |
|---|---|---|
| ℞ | Saltpetre | 1 part. |
| | Sugar | 2 parts. |
| | Glycerine | 16 parts. |

Methilic alcohol, enough to dissolve these ingredients.

Very little is used. After remaining a few weeks in that liquid, the joints were flexible and odorless. He had used benzine to destroy the fat in some cases.

Dr. Park had devoted many months to these researches, which will be highly satisfactory to every anatomist.

Dr. E. Fletcher Ingals read an abstract of a paper on

LARYNGEAL GROWTHS.

Up to twenty years ago, there had not been over seventy cases of this kind discovered, and those had, most of them, been found in the dead. But since that time thousands of cases had been found in the living. All sorts of tumors may be found in the larynx that are found elsewhere in the body, about in the following proportion: 75 per cent. are papillar growths, 25 per cent. fibrous, the rest were fibro-cellular, adenoid, etc., and malignant growths. It had been his misfortune to see more than his share of this last class, for he had treated three cases, which will be found with the following:

Case I. A man 63 years of age had a fibroid tumor at the upper part of the trachea. Had experienced hoarseness for seven years. Had been worse for three months. Difficulty of speech and dyspnœa. The tumor was a large pyriform growth, pressing on the vocal cords, 8 mm. in diameter. Patient did not consent to an operation.

II. In the case of a physician, a small tumor had grown on the left ventricular band, the size of a split pea, hiding the left vocal cord. A first attempt at removal did not succeed. Another trial will soon be made.

III. A. B. A sessile papilloma on the lower surface of left vocal cord, 5 mm. in diameter. It was removed by McKinsie's forceps, and patient was cured.

IV. A man 69 years old. A growth was found in the larynx, which had caused hoarseness, severe dyspnœa, and other symptoms, for ten years. A small piece of the tumor had been removed and examined under the microscope. It was pronounced malignant. It was removed repeatedly during some weeks, but a large swelling formed in the arytenoid fold, which sloughed, but could not be removed. Patient refused to have tracheotomy performed. He died after a few days.

V. A female, æt. 25. Had had polypi in the larynx ten years ago in Germany, where a surgeon had removed them. A few months ago she had the sensation of a bolus in the throat. On examination a growth the size of a small pea was found attached to one of the vocal cords. It was destroyed by nitrate of silver. A larger tumor was also fouud in her larynx, and a part of it removed.

VI. M. E. Began to experience some dyspnœa some months ago. The vocal cords were congested. A pedunculated papillary growth $\frac{1}{2}$ inch long hung in the sub-glottic space, having its point of attachment at the upper part of the posterior commissure of the vocal cord. It has not yet been removed.

VII. In this case a tumor the size of a pea had been removed from the right vocal cord, followed by perfect recovery.

VIII. A male, æt. 24, had had chronic laryngitis. Had pleurisy three months ago. Had also had pneumonia and remittent fever. Patient was probably tubercular. A small papillary growth of the vocal cords was destroyed with carbolic acid and solution of morphia. Tannic acid and glycerine were also applied, and the nitrate of silver used. Inhalations of tr. of benzoine were given, and cod liver oil administered. The right lung was affected at the apex. Patient gained three pounds while under treatment. He afterward went to Texas, where he died suddenly three weeks ago.

IX. T. N., æt. fifty-nine, had complained of pain and dyspnœa for four months. A nodular growth 2 mm. in size was found attached to the right ventricular band, the vocal cords not being involved. It was not removed. Two months later ulceration of the throat took place, and Dr. Ingals saw the patient when he had been unable to swallow for ten days on account of regurgitation into the larynx. It was evidently a cancer. The suffering was great. The Doctor did not think excision of the larynx should be performed in such cases, and he had met a few other ones, because the life is not likely to be prolonged thereby. Dr. Ingals said that the removal of growths from the larynx is not painful, but that it is very uncomfortable to the patient. A warm compress was held around the neck for thirty-six hours after each operation, to prevent inflammation. There was no risk of tearing the vocal cords in removing small growths. He had been so unfortunate as to tear the healthy mucous membrane, where it was not intended, once, but no ill results occurred. Papilloma should not be removed where they seem harmless, because of their liability to turn into malignant growths.

X. A large malignant growth on the side of the larynx came under his observation. He has delayed its removal till now, because there will be hæmorrhage, which he will control with a peculiar electrode which he has contrived.

Dr. Ingals does not believe, as some do, that laryngeal growths depend on syphilis or tuberculosis. H. D. V.

